# Seroprevalence, distribution, and risk factors for human leptospirosis in the United States Virgin Islands

**DOI:** 10.1371/journal.pntd.0010880

**Published:** 2022-11-15

**Authors:** Aileen Artus, Ilana J. Schafer, Caitlin M. Cossaboom, Dana L. Haberling, Renee Galloway, Graham Sutherland, A. Springer Browne, Joseph Roth, Valicia France, Hannah M. Cranford, Kristine J. Kines, Justine Pompey, Brett R. Ellis, Henry Walke, Esther M. Ellis, Leptospirosis Serosurvey Investigation Team

**Affiliations:** 1 Division of High Consequence Pathogens and Pathology, National Center for Emerging and Zoonotic Infectious Diseases, U.S. Centers for Disease Control and Prevention, Atlanta, Georgia, United States of America; 2 Epidemic Intelligence Service, U.S. Centers for Disease Control and Prevention, Atlanta, Georgia, United States of America; 3 U.S. Virgin Islands Department of Health, U.S. Virgin Islands, United States of America; 4 Division of Local and State Readiness, U.S. Centers for Disease Control and Prevention, Atlanta, Georgia, United States of America; 5 Division of Preparedness and Emerging Infections, U.S. Centers for Disease Control and Prevention, Atlanta, Georgia, United States of America; University of Connecticut Health, UNITED STATES

## Abstract

**Background:**

The first documented human leptospirosis cases in the U.S. Virgin Islands (USVI) occurred following 2017 Hurricanes Irma and Maria. We conducted a representative serosurvey in USVI to estimate the seroprevalence and distribution of human leptospirosis and evaluate local risk factors associated with seropositivity.

**Methodology/Principal findings:**

A stratified, two-stage cluster sampling design was used and consisted of three island strata and random selection of census blocks and then households. All eligible members of selected households were invited to participate (≥5 years old, resided in USVI ≥6 months and ≥6 months/year). Household and individual-level questionnaires were completed, and serum collected from each enrolled individual. Microscopic agglutination test serology was conducted, and bivariate and logistic regression analyses completed to identify risk factors for seropositivity.

In March 2019, 1,161 individuals were enrolled from 918 households in St. Croix, St. Thomas, and St. John. The territory-wide weighted seroprevalence was 4.0% (95% CI:2.3–5.7). Characteristics/exposures independently associated with seropositivity using logistic regression included contact with cows (OR: 39.5; 95% CI: 9.0–172.7), seeing rodents/rodent evidence or contact with rodents (OR: 2.6; 95% CI: 1.1–5.9), and increasing age (OR: 1.02; 95% CI: 1.002–1.04); full or partial Caucasian/White race was negatively correlated with seropositivity (OR: 0.02, 95% CI: 0.04–0.7). Bivariate analysis showed self-reported jaundice since the 2017 hurricanes (pRR: 5.7; 95% CI: 1.0–33.4) was associated with seropositivity and using a cover/lid on cisterns/rainwater collection containers (pRR: 0.3; 95% CI: 0.08–0.8) was protective against seropositivity.

**Conclusions/Significance:**

Leptospirosis seropositivity of 4% across USVI demonstrates an important human disease that was previously unrecognized and emphasizes the importance of continued leptospirosis surveillance and investigation. Local risk factors identified may help guide future human and animal leptospirosis studies in USVI, strengthen leptospirosis public health surveillance and treatment timeliness, and inform targeted education, prevention, and control efforts.

## Author summary

We established that in 2019, four percent of the population in the U.S. Virgin Islands (USVI) had evidence of previous exposure to *Leptospira* bacteria. There were no human cases of leptospirosis documented in USVI before the 2017 hurricanes, however due to the prevalence of the disease in other Caribbean localities, the post-hurricane cases, and the evidence of exposure demonstrated in this investigation, it is likely that leptospirosis has been a previously unrecognized problem in the territory. We demonstrated that contact with rodents or their body fluids or seeing rodents/evidence of rodents, contact with cows, increasing age, and not being part or full Caucasian/White race are independently associated with previous exposure to *Leptospira* spp. in USVI. These findings highlight the importance of continuing leptospirosis surveillance in USVI, considering leptospirosis as a diagnosis in patients with acute febrile illness and/or compatible exposures, and further investigating the epidemiology, ecology, and clinical features of leptospirosis in USVI. Our investigation also supports the need to investigate leptospirosis, and consider it a potential diagnosis, in locations with environmental conditions the bacteria favor (tropical and subtropical areas), even if leptospirosis has not been previously documented in those areas, and especially if it has not been previously investigated.

## Introduction

In the aftermath of two Category 5 hurricanes, Irma and Maria, in 2017, three patients from the United States Virgin Islands (USVI) were diagnosed with leptospirosis—the first human cases of leptospirosis ever documented in the territory [1]. Before 2017, the only available evidence of leptospirosis in USVI was a study published in 1992 demonstrating seropositivity among sheep and goats on the island of St. Croix [2]—there are no known investigations into human leptospirosis before 2017.

Leptospirosis is a widespread zoonotic disease, caused by bacteria in the genus *Leptospira*, with more than 300 pathogenic serovars identified [3]. Worldwide, over one million cases and close to 60,000 deaths are estimated each year [4]. While it is found globally, leptospirosis is most prevalent in tropical climates, with the Caribbean region having one of the highest estimated annual case incidences, 51 cases per 100,000 with 3 deaths per 100,000, and resource-poor areas are heavily impacted [4,5]. *Leptospira* spp. are spread in the urine of infected animals, with many implicated mammalian reservoirs and incidental hosts [6]. People are exposed to the bacteria either through direct urine-contact, or through environmental contact with urine-contaminated fresh water or mud. Bacterial transmission occurs through mucous membranes, skin abrasions, drinking contaminated water, and is suspected to occur through waterlogged skin [7]. *Leptospira* spp. can survive in fresh water and wet soil for weeks to months in appropriate environmental conditions [7,8]. As demonstrated in 2017, outbreaks can occur after extreme weather events like hurricanes and floods [1,9–14].

Diagnosing and treating patients with leptospirosis, a potentially fatal disease, as early as possible in the course of illness is extremely important as early antibiotic therapy may decrease the duration and severity of disease [15–17]. However, patients with leptospirosis are often mis-diagnosed, as the symptoms are similar to many other acute febrile diseases such as influenza and diseases commonly seen in the Caribbean and other tropical climates like dengue and Zika [17–22]. In addition, a lack of disease awareness by the public and clinicians and absence of adequate diagnostic test availability in some locations can contribute to under-diagnosis. While the majority of people infected with *Leptospira* spp. are asymptomatic, those that develop symptoms can have illness ranging from non-specific influenza-like illness with symptoms including fever, headache, myalgia, and gastrointestinal symptoms, to more severe illness with multi-organ dysfunction that may include renal failure, liver damage, meningitis, and hemorrhage. In general, approximately 5–15% of patients who develop severe illness die, however case-fatality rates can reach >50% with more severe disease manifestations such as Severe Pulmonary Hemorrhagic Syndrome [7,23].

Although the first reported cases of human leptospirosis in USVI were diagnosed in the months following the 2017 hurricanes, it is likely cases occurred in USVI well before that time based on the geographic region and climate, endemicity of leptospirosis in other Caribbean islands [4,10,24–26], and evidence of *Leptospira* spp. exposure in animals in USVI [2,27]. Therefore, a human leptospirosis serosurvey representative of the three largest inhabited islands, St. Croix, St. Thomas, and St. John, was conducted in March 2019 to contribute to public health surveillance by estimating the seroprevalence and distribution of leptospirosis in USVI and evaluating local risk factors associated with leptospirosis seropositivity, in order to determine the public health importance of this disease in USVI and inform future prevention and control efforts.

## Methods

### Ethics statement

This project was given a non-research determination by the CDC National Center for Emerging and Zoonotic Diseases Human Subjects Team (#021919AA) because it was a public health surveillance activity. Therefore, Institutional Review Board review was not required. The use of verbal consent by participants was authorized by the Human Subjects Team and was documented by indicating in the individual’s questionnaire that verbal consent was given.

### Sample size and population

From March 4–30, 2019, we performed a cross-sectional serosurvey of the three largest inhabited islands of USVI: St. Croix, St. Thomas (including Water Island), and St. John. Eligible individuals were USVI residents who were ≥5 years at the time of sampling, had resided in USVI for at least 6 months total, and on average resided in USVI at least six months per year. We calculated a minimum overall sample size of 1,174 individuals based on an estimated 3% leptospirosis seroprevalence in USVI; 2% margin of error; an alpha of 0.05; a design effect of 2; and replacement sampling with an estimated 95% completion rate to account for unusable samples or data. Sample size by island was made proportional to the 2010 U.S. Decennial Census island population size [28]: 552 in St. Croix, 575 in St. Thomas, and 47 in St. John.

### Sampling design and household selection

A stratified, two-stage cluster sampling design was utilized: stratification by island and clustering by census blocks and then households. Census blocks with zero population or consisting mostly of national park land were excluded. Based on the proportional island strata, a 2017 post-hurricane survey estimate of 1.9 people per household, and an intention to capture a wide distribution of people across the islands, we targeted a minimum of 37, 38, and 4 census blocks in St. Croix, St. Thomas, and St. John, respectively. Census blocks were randomly selected and sampled in chronologic order of selection until target sample size per island was reached. Households were systematically selected and up to eight households per census block were enrolled—fewer than eight were enrolled if the census block had fewer than eight households or if every household in the census block was visited but eight could not be enrolled.

Field teams selected the house closest to the census block centroid as the first selected household and then moved through the census block in a serpentine fashion (up one side of the street and down the other) and consistent direction to select the next households using an interval number sequence specific to the census block (total housing units divided by eight). The interval number used for selection ensured even distribution of selected households throughout the census block. Selected households not enrolled were sequentially replaced by the next adjacent house. Field teams continued to loop through the census block until eight households were enrolled or all houses in the census block had been visited. Households visited in the sampling process were categorized as either enrolled, refused, or unavailable. Unavailable households included those where there was no answer, the selected household was inaccessible to the field team for safety or logistical reasons, the household agreed to participate but no blood sample was successfully collected, or the household was ineligible (no eligible members or no members ≥18 years of age present).

### Household and participant enrollment

An eligible household was enrolled if ≥1 eligible household member agreed to participate. All eligible household members were invited to participate and were enrolled if they agreed to both have a blood sample collected and verbally complete a questionnaire. Verbal consent from participants ≥18 years old and verbal assent with parent/guardian permission from participants <18 years old was obtained prior to enrollment. If a revisit was requested to enroll additional eligible household members not present, teams attempted to return to households up to two additional times, as logistics allowed, to attempt to enroll those individuals.

### Questionnaire administration

Questionnaires were verbally administered using the Epi-Info 7 mobile application on tablets. A participating representative of the household was interviewed to complete a household questionnaire including information on household-level demographics; drinking water sources and treatment methods; animals owned or seen in the house or on the land; house or land flooding from heavy rains; and damage to the home and changes in drinking water source(s) due to the 2017 hurricanes (S1 Appendix). Neighborhood type was subjectively assigned by interviewers as rural, urban, or suburban at the time of survey–using the following guidelines: urban if within a major city, suburban if somewhat populated and primarily residential, and rural if sparsely populated with unpaved roads. Each participant completed an individual questionnaire including information on individual-level demographics of age, sex, race, ethnicity, and occupation; risk factors for leptospirosis including water and animal exposures in the previous year; and post-hurricane behaviors/activities and illness (S2 Appendix).

### Specimen collection and laboratory testing

Each participant had approximately 5 ml of blood collected into a serum-separator tube which was centrifuged within a few hours of collection and then transported to the US Virgin Islands Public Health Laboratory. Sera were shipped frozen and tested at the CDC Bacterial Special Pathogens Branch Laboratory for anti-*Leptospira* spp. antibodies by the microscopic agglutination test (MAT), using an antigen panel of 20 serovars (S1 Table) representing 17 serogroups [29]. A positive test result was defined as a titer of 1:100 or greater to any antigen in the panel.

### Statistical analysis

All categorical demographics and risk factors are expressed as weighted percentages with 95% confidence intervals (CI). Rao-Scott Chi-Square test and Taylor series variance estimates were used to determine any association between a positive test result and participant location, demographics, and risk factors—prevalence risk ratios (pRR) and corresponding 95% CI are presented. The reference category for all comparisons is the “No” or “False” value unless a reference value is otherwise indicated. Continuous variables are expressed as means or medians as noted with 95% CI using least squares Taylor series linearization variance estimates. A T-test was used to test differences in means by serologic status. All data analyses were weighted by the probability of selection of the census block and household by island and adjusted to match the distribution of age and sex by island using the US 2010 Decennial Census [28].

Free text values from “other” responses were reassigned to an existing pre-defined response when appropriate, including for occupation, drinking water source, water protection and treatment, animals owned or seen in the house or land, animal contact, and fresh water or mud contact. The total number of remaining, non-reassigned “other” responses are presented but not analyzed by participant serology status since some responses were not legitimate based on review. New variables were created for commonly repeated responses in free text fields for further analysis. Some response values were combined to create additional variables for analysis: local bottled water and stateside bottled water usage were combined to create an overall bottled water category, cistern water and non-cistern collected rainwater were combined as one collected rainwater category; boiling, chlorination and UV light as one water treatment category; and seen/owned rodents in home or property and contact with rodents as one category.

Logistic regression was used for analyzing independent associations with seropositivity; weighted estimates for odds ratios and corresponding 95% CIs to account for sampling design and corresponding p-values are presented for these results. The models were analyzed for collinearity, interactions, and confounding. We considered sex as a potential confounder, however its inclusion made no significant difference to point estimates and was not included in the final model. All statistical analyses incorporated the sample design using SAS/STAT software, Version 9.4 for Windows, Cary, NC. Statistical significance was determined at p<0.05.

### Spatial distribution mapping

The geographic distribution of total collected samples and positive samples by census block were mapped using ESRI ArcGIS (v 10.8.1). A choropleth map and graduated symbols were used to represent the number of samples collected and the number of positive samples, respectively. Graduated symbols were plotted using census block centroid coordinates. To protect participant privacy in small census blocks selected for sampling (<5 household units based on the 2010 U.S. Decennial Census), we used the dissolve tool to merge small census blocks with an adjacent census block to create a merged polygon with ≥5 households. The combination with the least number of merged census blocks and the least number of households to reach ≥5 households in the merged polygon was prioritized.

## Results

**Fig 1:**
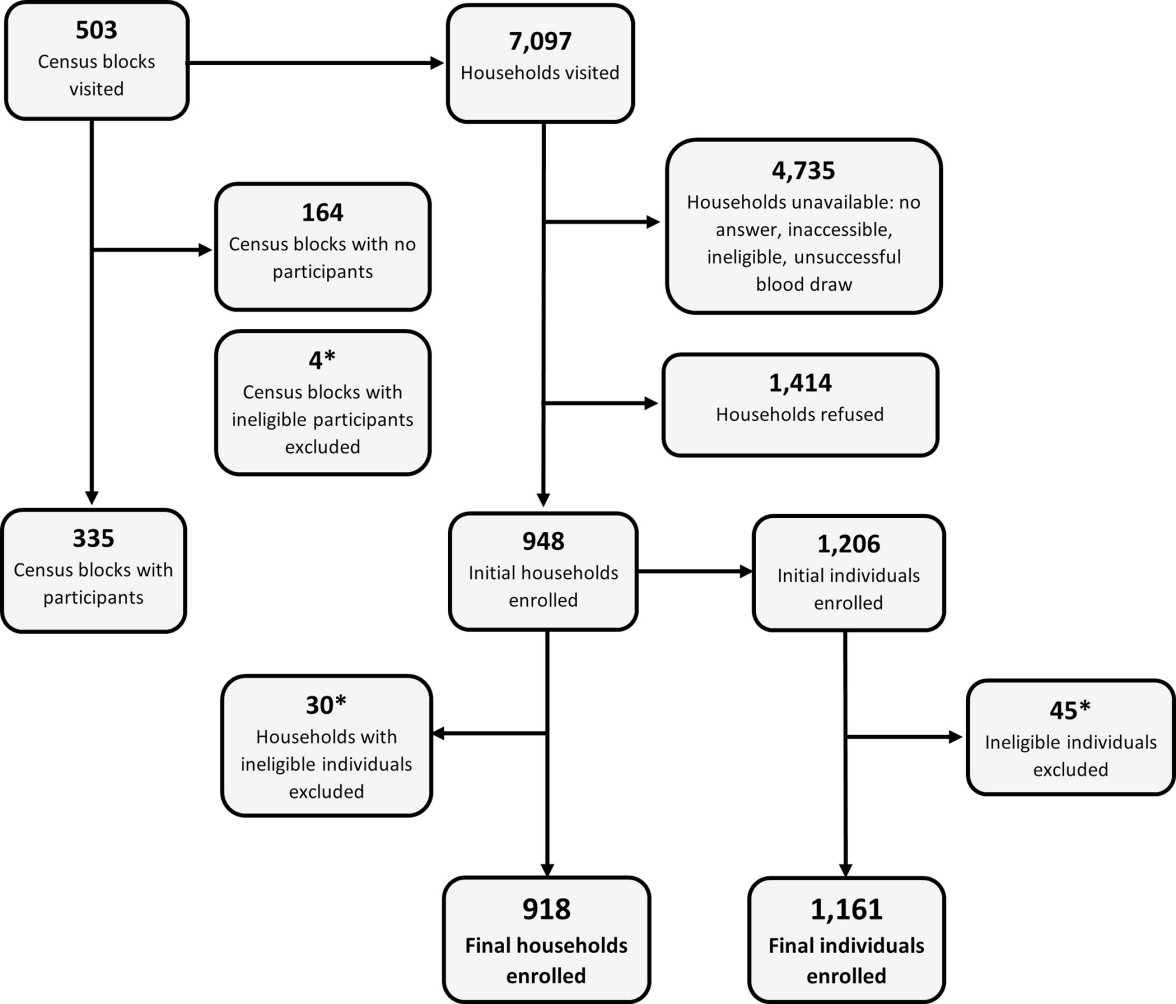
Sampling and Enrollment Diagram for the Leptospirosis Serosurvey in USVI, March 2019. * Post-survey preliminary data cleaning and analysis revealed 45 participants who did not meet the eligibility criteria and were enrolled in error, resulting in their exclusion from final analysis. Thirty households and four census blocks with only ineligible individuals enrolled were removed from the final counts.

A total of 1,161 participants from 918 households were successfully enrolled in the serosurvey. During March 4–30, 2019, 7,097 households were visited in St. Croix, St. Thomas (including Water Island), and St. John: 1,414 (19.9%) households refused participation; and 4,735 (66.7%) households were unavailable. Across the territory, field teams visited 503 census blocks (St. Croix: 224, St. Thomas: 253, St. John: 26). Initially, 1,206 individual participants from 948 (13.4%) households were enrolled in the survey. Post-survey preliminary data cleaning and analysis revealed 45 participants who did not meet the eligibility criteria (had lived in USVI <6 months) and were enrolled in error, resulting in their exclusion from final analysis (Fig 1). Among the 2,362 households that a field team had contact with that were eligible for participation there was a 40.1% enrollment rate and a 59.9% refusal rate—this excludes households that agreed to participate but from which a blood draw was unsuccessful. Enrollment and refusal rates were similar between islands—refusal rates were 54.8%, 64.1%, and 55.4% for St. Croix, St. Thomas, and St. John, respectively.

**Fig 2:**
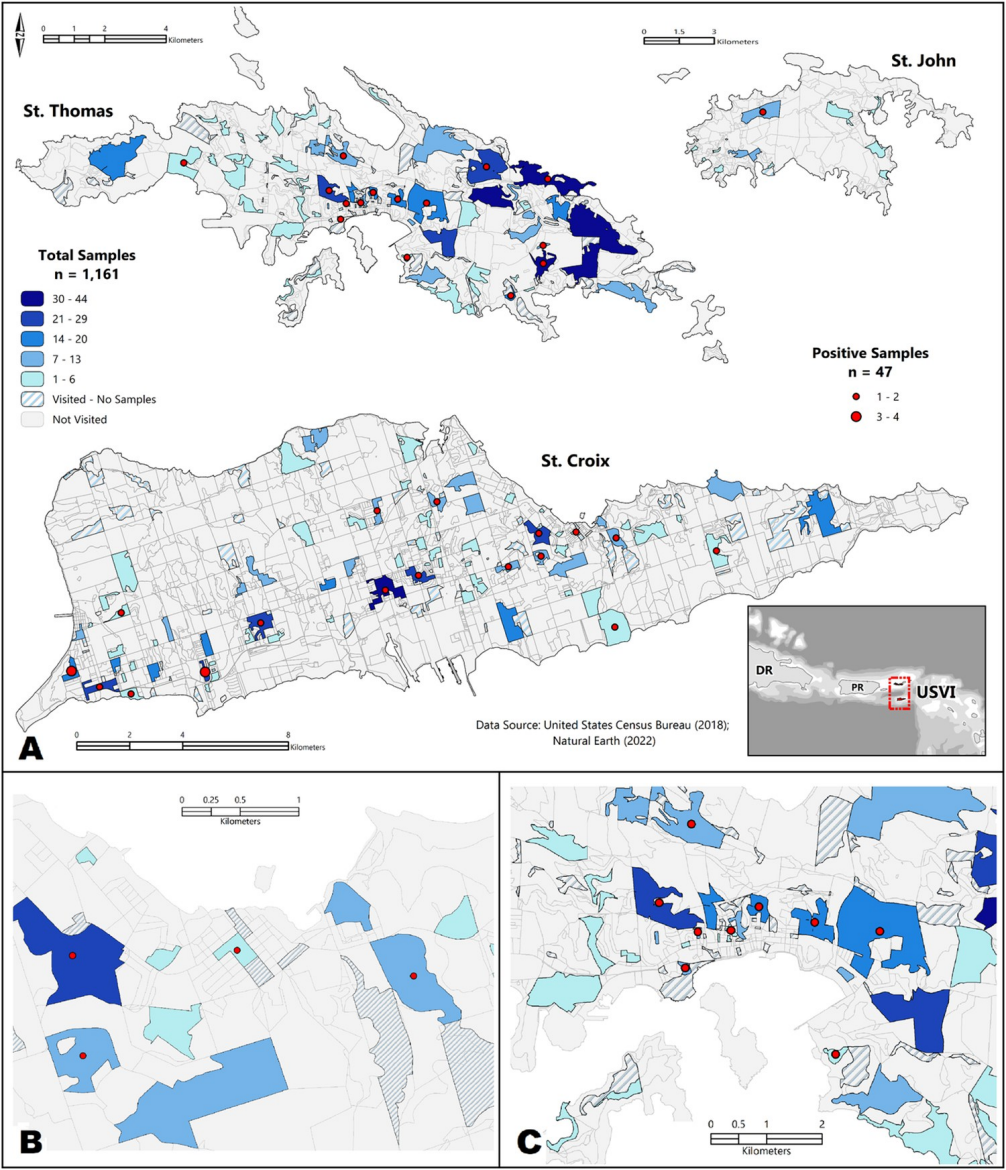
Distribution of collected samples and seropositive results by census block in the USVI leptospirosis serosurvey, March 2019. Panel A) Three main islands of USVI; Panel B) Magnification of Christiansted, St. Croix; Panel C) Magnification of Charlotte Amalie, St. Thomas. Sources: United States Census Bureau (FIPS Code: 78, https://www2.census.gov/geo/tiger/TIGER2018/TABBLOCK/); Natural Earth Data–country boundaries (https://www.naturalearthdata.com/downloads/10m-cultural-vectors/), bathymetry (https://www.naturalearthdata.com/downloads/10m-physical-vectors/).

Among the 1,161 participants enrolled and eligible for inclusion, 47% (n = 547) were from St. Croix, 49% (n = 566) from St. Thomas, and 4% (n = 48) from St. John. Participants were enrolled from 335 (66.6%) census blocks (St. Croix: 156, St. Thomas: 163, St. John: 16) (Fig 2). A mean of 2.8 households per census block (range: 1–8 households) and 1.3 individuals per household (range: 1–6 individuals) participated in the serosurvey across the territory.

The following descriptions of serosurvey participants are in weighted percentages. Fifty percent of the participants were female (95% CI: 47–55). Participant ages ranged from 5–94 years old, with a mean age of 42 years (95% CI: 39–44). Fifty-eight percent (95% CI: 53–63) of participants were aged 20–59 years, 22% (95%CI: 18–26) were 60 years and over, and 20% (95% CI: 15–25) were under 20 years of age. The majority (61%, 95% CI: 55–67) of participants reported being full or part African American/Black, followed by full or part Caucasian/White (18%, 95% CI: 13–24). Few participants (0.8%, 95% CI: 0.2–1.3) reported being full or part American Indian/Alaska Native, Asian, or Native Hawaiian/Pacific Islander race. Four percent (95% CI: 3–6) of participants reported a different race from standard race categories. Seventeen percent (95% CI: 12–22) of participants reported being of Hispanic or Latino ethnicity. Based on eligibility criteria and participant response to demographic questions, participant sex, age, race, and ethnicity distribution appears similar to the USVI demographics in the 2010 US Decennial Census. Table 1 describes the demographic and travel characteristics of serosurvey participants including raw counts and weighted percentages by serologic result.

**Table 1:** Demographic and Travel Characteristics of Leptospirosis Serosurvey Participants in USVI by Serology Result, March 2019.

Characteristic	Total, N*	Seropositive		Seronegative		Prevalence Risk Ratio (95% CI)	P-value^^^^
		N*	Weighted Percent^^^ (95% CI)	N*	Weighted Percent^^^ (95% CI)		
**Total participants**	1161	47	4.0 (2.3–5.7)	1114	96.0 (94.3–97.7)	--	--
**Island**							
St. Croix	547	26	4.3 (1.4–7.3)	521	95.7 (92.7–98.6)	reference	
St. Thomas^**†**^	566	19	3.6 (1.7–5.5)	547	96.4 (94.5–98.3)	0.8 (0.3–2.0)	0.7
St. John	48	2	5.4 (0–13.0)	46	94.6 (87.0–100)	1.3 (0.3–5.9)	0.8
**Age group (years)**							
5–19	70	2	3.8 (0–10.0)	68	96.2 (90.0–100)	1.2 (0.2–7.5)	0.8
20–39	203	8	3.2 (0.8–5.6)	195	96.8 (94.4–99.2)	reference	
40–59	353	13	3.3 (0.9–5.8)	340	96.7 (94.2–99.1)	1.0 (0.4–3.0)	0.9
60–79	458	21	6.1 (2.9–9.4)	437	93.9 (90.6–97.1)	1.9 (0.8–4.8)	0.1
≥80	75	3	6.3 (0–14.7)	72	93.7 (85.3–100)	2.0 (0.4–9.0)	0.4
**Sex**							
Male	499	31	4.3 (2.3–6.4)	468	95.7 (93.6–97.7)	1.2 (0.5–2.7)	0.7
Female	659	16	3.7 (1.1–6.3)	643	96.3 (93.7–98.9)	reference	
**Race** **							
African American or Black	714	29	3.7 (2.0–5.4)	685	96.3 (94.6–98.0)	0.8 (0.3–2.0)	0.6
American Indian or Alaska Native	9	2	11.2 (0–29.7)	7	88.8 (70.3–100)	2.8 (0.5–15.4)	0.2
Asian	8	0	--	8	100 (100–100)	--	--
Caucasian or White	200	6	1.6 (0–3.1)	194	98.4 (96.9–100)	0.3 (0.1–1.0)	0.04
Native Hawaiian or Pacific Islander	1	0	--	1	100 (100–100)	--	--
Hispanic or Latino	181	6	7.0 (0–14.3)	175	93.0 (85.7–100)	2.1 (0.7–6.1)	0.2
Other race^**††**^	70						
**Occupation** **							
Maintenance; repair; construction; building/grounds cleaning staff	136	6	2.8 (0.1–5.6)	130	97.2 (94.5–99.9)	0.7 (0.2–1.9)	0.4
Office worker	111	2	1.2 (0–3.3)	109	98.8 (96.7–100)	0.3 (0.04–1.7)	0.1
Tourism/hospitality staff	73	0	--	73	100 (100–100)	--	--
Student	69	2	4.5 (0–11.8)	67	95.5 (88.2–100)	1.2 (0.2–6.1)	0.9
Healthcare provider	48	3	5.4 (0–12.7)	45	94.6 (87.3–100)	1.4 (0.3–5.8)	0.7
Law enforcement officer; firefighter	34	2	2.9 (0–7.1)	32	97.1 (92.9–100)	0.7 (0.2–3.3)	0.7
Food service worker^**‡**^	30	1	2.6 (0–7.7)	29	97.4 (92.3–100)	0.6 (0.08–4.9)	0.7
Retail worker^**‡**^	28	2	10.8 (0–24.4)	26	89.2 (75.6–100)	2.8 (0.8–10.3)	0.1
Educator^**‡**^	25	1	4.9 (0–14.4)	24	95.1 (85.6–100)	1.2 (0.2–9.0)	0.8
Housekeeping^**‡**^	13	1	6.0 (0–18.0)	12	94 (82–100)	1.5 (0.2–12.3)	0.7
Crop farmer	10	1	3 (0–9.2)	9	97.0 (90.8–100)	0.8 (0.1–6.1)	0.8
Livestock farmer	7	1	3.3 (0–10.9)	6	96.7 (89.1–100)	0.8 (0.1–8.7)	0.9
Fisherman	6	2	35.5 (0–79.5)	4	64.5 (20.5–100)	9.2 (2.3–36.6)	<0.001
Forestry; park worker; ranger	5	0	--	5	100 (100–100)	--	--
Landscaper^**‡**^	5	0	--	5	100 (100–100)	--	--
Military	3	0	--	3	100 (100–100	--	--
Veterinarian; vet or kennel staff	3	0	--	3	100 (100–100)	--	--
Plumber; sewer worker	2	0	--	2	100 (100–100	--	--
Slaughterhouse worker; meat inspector	1	0	--	1	100 (100–100)	--	--
Unemployed; retired	514	18	4.4 (1.8–7.0)	496	95.6 (93.0–98.2)	1.1 (0.5–2.5)	0.8
Other occupation^**††**^	64						
**Live outside USVI for part of year (≤6 months per year)**	89	5	6.1 (0–13.0)	84	93.9 (87.0–100)	1.6 (0.5–5.3)	0.5
**Traveled outside USVI in the last year**	711	29	3.5 (2.0–5.0)	682	96.5 (95.0–98.0)	1.5 (0.6–3.9)	0.4

*Raw numbers, not weighted

**The reference category for pRR and p-value comparisons is the”No” or “False” value for each characteristic

^Estimates expressed as weighted percentages with 95% confidence intervals (CI)

^^Chi-square test used for testing differences between categorical variables

**†**Includes Water Island

**††**Total counts for “Other” categories are presented but not estimates by serology status since some responses were found to be either not legitimate or difficult to interpret

**‡**Category was created by extracting responses from “Other” free text fields

—Undefined weighted percent or pRR where there are no cases in one or more comparison groups

Most participants resided full time in USVI (92%, 95% CI: 90–95) and for an average of 25 years (95% CI: 23–27, range: 1–94 years). Of the 8% (95% CI: 5–10) who reported partially residing outside of USVI, an average of 7 months per year (95% CI: 6.7–7.9) was spent in USVI. Seventy percent (95% CI: 65–75) of participants reported traveling outside of the territory in the last year, of which 40% (95% CI: 33–47) reported traveling to a Caribbean island and about half (51%; 95% CI: 45–58) travelled within the 50 U.S. states only.

A territory-wide weighted 4.0% seropositivity (n = 47; 95% CI: 2.3–5.7) for anti-*Leptospira* spp. antibodies was demonstrated (Table 1). Forty-seven individual seropositive participants had observed MAT titers that ranged from 1:100 to 1:3200. Seventeen percent (n = 8) had a highest titer of ≥1:400, of which all were single serogroup reactions. One participant had a highest titer of 1:3200 to serogroup Pyrogenes, two had a 1:1600 highest titer to serogroups Australis and Ballum, three had 1:800 highest titers to serogroups Djasiman (n = 1) and Icterohaemorrhagiae (n = 2), and two had 1:400 highest titers to serogroups Icterohaemorrhagiae and Pyrogenes (S2 Table). Among the 83% of seropositives (n = 39) with a highest titer of 1:100 to 1:200, 20% (n = 8) reacted to multiple serogroups. Among all seropositive participants, serogroup Icterohaemorrhagiae was the most common highest reacting serogroup (45%, n = 21), followed by serogroup Australis (26%, n = 12). Highest titers were also observed to serogroups Canicola (n = 7), Pyrogenes (n = 5), Tarrasovi (n = 4), Autumnalis (n = 2), Bataviae (n = 2), Djasiman (n = 2), Ballum (n = 1), and Sejroe (n = 1).

Characteristics significantly associated with seropositivity by bivariate analysis included residing in an urban neighborhood (pRR: 3.0; 95% CI: 1.0–9.0, p = 0.03), fishing as an occupation (pRR: 9.2; 95% CI: 2.3–36.6; p<0.001), seeing rodents or evidence of rodents in or around the house or land (pRR: 2.6; 95% CI: 1.2–6.0; p = 0.01), seeing deer in or around the house or land (pRR: 3.2; 95% CI: 1.0–10.0; p = 0.04), contact with rodents or their body fluids (pRR: 8.0; 95% CI: 1.9–34.1, p = 0.002), contact with cows or their body fluids (pRR: 14.0; 95% CI: 5.6–35.2; p<0.001), and having been ill with jaundice since the 2017 hurricanes (pRR: 5.7; 95% CI: 1.0–33.4; p = 0.04) (Tables 1 and 2). Characteristics significantly protective against seropositivity by bivariate analysis included being full or part Caucasian/White race (pRR: 0.3; 95% CI: 0.1–1.0; p = 0.04), using a cover or lid for cisterns and non-cistern rainwater collection containers (pRR: 0.3; 95% CI: 0.08–0.8; p = 0.009), and seeing bats in or around the house or land (pRR: 0.1; 95% CI: 0.01–1.1; p = 0.02) (Tables 1 and 2). There was no difference in seropositivity when comparing between islands, sex, and age categories. Seropositive participants had a mean age of 45 years (95% CI 34–57; range 5–87 years).

**Table 2:** Household and Individual Exposures and Risk Factors of Leptospirosis Serosurvey Participants in USVI by Serology Result, March 2019.

Risk factor	Total, N	Seropositive		Seronegative		Prevalence Risk Ratio (95% CI)	P-value^^
		N*	Weighted Percent^ (95% CI)	N*	Weighted Percent^ (95% CI)		
**Neighborhood type** ^**†**^							
Rural	187	6	2.1 (0.1–4.0)	181	97.9 (96.0–99.9)	reference	
Urban	234	14	6.2 (2.7–9.8)	220	93.8 (90.2–97.3)	3.0 (1.0–9.0)	0.03
Suburban	740	27	3.8 (1.3–6.2)	713	96.2 (93.8–98.7)	1.8 (0.6–5.5)	0.3
**Household drinking water, ever used** **							
Bottled water (local or stateside)	1161	47	4.0 (2.3–5.7)	1114	96.0 (94.3–97.7)	--	--
Piped water (WAPA)	196	13	4.1 (1.3–6.9)	183	95.9 (93.1–98.7)	1.0 (0.4–2.5)	0.9
Cistern water	517	19	2.5 (1.1–3.9)	498	97.5 (96.1–98.9)	0.5 (0.2–1.1)	0.08
Non-cistern rainwater container	83	3	2.9 (0–6.1)	80	97.1 (93.9–100)	0.7 (0.2–2.4)	0.6
Personal well	43	5	7.4 (0–15.4)	38	92.6 (84.6–100)	1.9 (0.6–6.1)	0.3
Saltwater (by reverse osmosis)^**‡**^	5	0	--	5	100 (100–100)	--	--
**Household drinking water, primary source**							
Bottled water (local or stateside)	1008	39	3.9 (2.1–5.8)	969	96.1 (94.2–97.9)	0.9 (0.3–2.4)	0.8
Piped water (WAPA)	18	1	2.8 (0–8.5)	17	97.2 (91.5–100)	0.7 (0.08–5.7)	0.7
Cistern water	127	7	5.0 (0.4–9.5)	120	95.0 (90.5–99.6)	1.3 (0.5–3.6)	0.6
**Protection and treatment of cistern and non-cistern collected rainwater** **							
Cover/lid	505	19	2.3 (0.9–3.6)	486	97.7 (96.4–99.1)	0.3 (0.08–0.8)	0.009
Filtration system	334	13	2.9 (1–4.9)	321	97.1 (95.1–99.0)	1.5 (0.5–4.7)	0.4
Boiling	224	7	2.2 (0.5–4.0)	217	97.8 (96–99.5)	0.9 (0.3–2.6)	0.9
Chlorination	360	11	1.4 (0.2–2.5)	349	98.6 (97.5–99.8)	0.4 (0.1–1.2)	0.08
UV light	83	3	2.4 (0–5.4)	80	97.6 (94.6–100)	1.0 (0.2–4.3)	>0.9
**Own/saw animals in house or land** **	949	41	4.4 (2.4–6.3)	908	95.6 (93.7–97.6)	1.9 (0.7–4.8)	0.2
Rodents or evidence of rodents	727	33	5.3 (2.8–7.8)	694	94.7 (92.2–97.2)	2.6 (1.2–6.0)	0.01
Dogs	684	32	4.5 (2.0–7.0)	652	95.5 (93.0–98.0)	1.4 (0.6–3.2)	0.5
Cows	21	2	5.4 (1.2–9.6)	19	94.6 (90.4–98.8)	1.3 (0.6–3.1)	0.5
Pigs	22	1	4.8 (0.3–9.2)	21	95.2 (90.8–99.7)	1.2 (0.5–3.1)	0.7
Horses	99	5	1.9 (0–4.0)	94	98.1 (96.0–100)	0.4 (0.1–1.5)	0.2
Goats/sheep	66	5	4.5 (0.4–8.5)	61	95.5 (91.5–99.6)	1.1 (0.4–3.0)	0.8
Deer^**‡**^	119	8	10.5 (0–21.6)	111	89.5 (78.4–100)	3.2 (1.0–10.0)	0.04
Mongoose^**‡**^	140	12	7.3 (2.1–12.4)	128	92.7 (87.6–97.9)	2.0 (0.9–4.7)	0.1
Bats^**‡**^	26	1	0.5 (0–1.6)	25	99.5 (98.4–100)	0.1 (0.01–1.1)	0.02
**Animal contact** ** ^¶^	616	22	3.7 (1–6.3)	594	96.3 (93.7–99.0)	0.8 (0.3–2.0)	0.7
Rodents^**‡**^	13	1	27.8 (0–66.4)	12	72.2 (33.6–100)	8.0 (1.9–34.1)	0.002
Dogs	583	21	3.8 (1.1–6.5)	562	96.2 (93.5–98.9)	0.9 (0.4–2.1)	0.8
Cows	25	6	42.3 (6.6–78.1)	19	57.7 (21.9–93.4)	14.0 (5.6–35.2)	<0.001
Pigs	23	3	9.2 (0–20.9)	20	90.8 (79.1–100)	2.4 (0.6–9.0)	0.2
Horses/donkeys	73	4	4.8 (0–10.4)	69	95.2 (89.6–100)	1.2 (0.3–4.2)	0.8
Goats/sheep	43	4	6.4 (0–14.7)	39	93.6 (85.3–100)	1.6 (0.4–6.4)	0.5
Deer^**‡**^	12	0	--	12	100 (100–100)	--	--
Mongoose^**‡**^	8	1	10.2 (0–30.3)	7	89.8 (69.7–100)	2.6 (0.3–19.1)	0.4
Bats^**‡**^	8	2	8.5 (0–23.1)	6	91.5 (76.9–100)	2.1 (0.4–12.3)	0.4
**Fresh water or mud contact** **	578	23	5.0 (1.8–8.3)	555	95 (91.7–98.2)	1.6 (0.7–3.8)	0.2
River or stream (running water)	86	4	2.9 (0–6.3)	82	97.1 (93.7–100)	0.7 (0.2–2.4)	0.6
Lake or pond (still water)	62	5	8.2 (0–16.8)	57	91.8 (83.2–100)	2.2 (0.7–6.7)	0.2
Untreated cistern water	281	11	3.2 (0.8–5.5)	270	96.8 (94.5–99.2)	0.8 (0.3–1.8)	0.5
Marsh or swamp	22	2	5.3 (0–13.0)	20	94.7 (87.0–100)	1.3 (0.3–6.1)	0.7
Mud/wet soil	386	17	5.7 (1.2–10.1)	369	94.3 (89.9–98.8)	1.7 (0.7–4.4)	0.2
Sewage water or run-off	115	7	5.5 (1–10.1)	108	94.5 (89.9–99.0)	1.4 (0.6–3.6)	0.4
Other fresh water^**††**^	22						
**Activities leading to fresh water or mud contact** **							
Bathing, wading, swimming, or walking barefoot	301	14	5.6 (0.8–10.3)	287	94.4 (89.7–99.2)	1.6 (0.6–4.2)	0.3
Boating, canoeing, or kayaking	70	3	3.2 (0–7.0)	67	96.8 (93.0–100)	0.8 (0.2–2.8)	0.7
Gardening or farming	498	22	6.0 (2–10.0)	476	94.0 (90.0–98.0)	2.0 (0.9–4.6)	0.08
Hiking	152	10	7.5 (0–14.9)	142	92.5 (85.1–100)	2.2 (0.7–6.7)	0.1
Other activities^**††**^^**§**^	52						
**Flooding in house or land during heavy rains**	336	21	4.0 (1.9–6.1)	315	96.0 (93.9–98.1)	1.0 (0.5–2.0)	>0.9
If yes, flooding occurred in the last year	218	16	4.3 (1.6–6.9)	202	95.7 (93.1–98.4)	1.3 (0.4–4.6)	0.7
**Exposures or outcomes related to the 2017 hurricanes** **							
Touched flood waters	476	25	3.8 (1.7–5.9)	451	96.2 (94.1–98.3)	1.0 (0.4–2.2)	0.9
Cleaned debris	816	37	4.5 (2.2–6.8)	779	95.5 (93.2–97.8)	2.0 (0.8–5.3)	0.1
Home was flooded	654	31	3.8 (2.2–5.5)	623	96.2 (94.5–97.8)	0.9 (0.3–2.4)	0.8
Home or property was damaged	704	28	3.6 (1.9–5.3)	676	96.4 (94.7–98.1)	0.9 (0.35–2.1)	0.8
Continued to live in home while damaged	568	23	3.9 (2.0–5.9)	545	96.1 (94.1–98.0)	1.3 (0.4–3.6)	0.7
Did not live in home but visited or worked on home while damaged	79	5	5.4 (0.1–10.7)	74	94.6 (89.3–99.9)	--	--
**Illness since the 2017 hurricanes** **							
Flu-like illness	403	16	5.0 (1.2–8.9)	387	95.0 (91.1–98.8)	1.4 (0.6–3.5)	0.4
Jaundice	7	1	22.6 (0–60.9)	6	77.4 (39.1–100)	5.7 (1.0–33.4)	0.04
Kidney failure	24	3	3.8 (0–9.4)	21	96.2 (90.6–100)	0.9 (0.2–4.5)	0.9
Hospitalized	72	6	6.9 (0–14.5)	66	93.1 (85.5–100)	1.8 (0.6–5.8)	0.3

*Raw numbers, not weighted

**The reference category for pRR and p-value comparisons is the “No” or “False” value of the characteristic

^Estimates expressed as weighted percentages with 95% confidence intervals (CI)

^^Chi-square test used for testing differences between categorical variables

^**†**^Subjectively assigned by interviewer based on observation of neighborhood type

^**††**^Total counts for “Other” categories are presented but not estimates by serology status since some responses were found to be either not legitimate or difficult to interpret

^**‡**^Category was created by extracting responses from “Other” free text fields

^¶^Types of contact include feeding, grooming, milking, cleaning up areas where the animals are housed, and contact with bodily fluid

^**§**^One person in the “other” response category for activities leading to fresh water or mud contact was seropositive. This person indicated they worked in irrigation ponds.

—Undefined weighted percent or pRR where there are no cases in one or more comparison groups

Characteristics and exposure factors independently associated with seropositivity by multivariate logistic regression included contact with cows or their body fluids (OR = 39.5, 95% CI = 9.0–172.7, p = <0.001); seeing rodents/evidence of rodents or contact with rodents or their body fluids (OR = 2.6, 95% CI = 1.1–5.9, p = 0.02); and increasing age (OR = 1.02, 95% CI = 1.002–1.04, p = 0.03). Reporting being full or part Caucasian/White race was protective against seropositivity (OR = 0.2, 95% CI = 0.04–0.7, p = 0.02) by multivariate logistic regression.

## Discussion

The demonstration of four percent leptospirosis seropositivity across USVI is a significant finding, as before Hurricanes Irma and Maria in 2017, the disease was previously unrecognized in people in USVI [1]. The serosurvey was conducted approximately 18 months after the hurricanes, however MAT-positive serology results do not indicate the timing of *Leptospira* spp. exposure and anti-*Leptospira* antibodies can persist for years, therefore it is difficult to determine whether seropositive persons were exposed before or after the 2017 hurricanes and they likely represent exposures from both time periods. Although there are no documented cases or published investigations of human leptospirosis in USVI before 2017, it is likely that undetected *Leptospira* spp. infections in people occurred before that time based on the previous detection of anti-*Leptospira* antibodies in animals in USVI [2,27], the tropical climate conducive to *Leptospira* spp. environmental survival, and the known endemicity of *Leptospira* spp. in other Caribbean islands [4,10,24–26].

There is limited information on seroprevalence of leptospirosis for the general human population in the Caribbean, as most published literature consists of testing among patients with acute febrile illness, and of the few serosurveys, many focus specifically on high-risk populations or a limited subset of the population. A seroprevalence investigation published in 1963 of patients and staff at medical facilities in Puerto Rico without evidence of an infectious disease demonstrated a 13.9% (268/1931) seroprevalence for anti-*Leptospira* spp. antibodies [30]. A 1980 household serosurvey conducted in urban and rural communities in Barbados and Trinidad demonstrated 18.5% (93/504) and 21.9% (111/508) seropositivity in each location respectively but used a lower MAT titer cutoff for positivity (1:50) than our investigation [31]. From 2009–2011, 442 healthy pregnant women from 10 Caribbean countries were tested and demonstrated 18.6% leptospirosis seroprevalence, using ELISA IgG rather than MAT [32]. Compared to these historical studies and as hypothesized in our sample size calculations, the seroprevalence demonstrated in USVI is lower; however, the design of our investigation in USVI was statistically representative of the entire territory unlike the locations represented in the historical studies, there is a large time difference in the investigations conducted, and serology testing methods and cutoffs differed, therefore the results are not entirely comparable. Also, unlike other Caribbean Islands, USVI does not have any natural large bodies of fresh water, an important potential source of exposures to *Leptospira* spp. in other locations.

The burden of leptospirosis in USVI may be under or over-represented by our cross-sectional finding of four percent seropositivity. On one hand, anti-*Leptospira* spp. agglutinating antibodies may stop being detectable as early as one year post-infection [33], and many people are seronegative by a few years post-infection [33,34], therefore the four percent seropositivity likely represents more recent exposures, not all previous exposures, and the true percentage of the population previously exposed is likely higher. Alternatively, this serosurvey was conducted one and a half years after Hurricanes Irma and Maria, which caused massive destruction and created an environment that led to a significant increase in high risk exposure types for leptospirosis, including standing floodwater, damaged houses, increased trash accumulation, and increased exposure to rodents and their urine. Therefore, the seropositivity observed was likely higher than would have been seen pre-hurricanes.

No significant difference was detected in the seropositivity of participants between islands; however, this survey was designed to estimate seropositivity across USVI and our sample size did not have adequate statistical power to detect a difference between islands. The 45 ineligible participants inadvertently enrolled that were removed during analysis did not affect the representativeness of our results; the calculated sample size accounted for up to a five percent loss for potentially unusable samples or data and the initial enrollment oversampled by 32, therefore only a 1.1% loss (n = 13) occurred. In addition, representativeness was ensured by adjusting the probability of selection using the 2010 US Census to obtain weighted estimates of prevalence.

The vast majority of seropositive individuals had low MAT titers, which was expected, as after initial titer peak in the one to two months post-infection [35,36], MAT titers can decline quickly, especially in the first year after infection [33,35]. The few participants with high MAT titers most likely had a more recent infection. One participant with a titer of 1:1600 measured during the serosurvey was retrospectively identified as a previously confirmed case of leptospirosis that had been sick and diagnosed with leptospirosis approximately three months before the serosurvey occurred, at which point a MAT titer of 1:12800 was measured. Serogroups identified by MAT remain useful for epidemiological and ecological investigative purposes, despite taxonomic classification moving from serologic towards genomic methods. The most common highest reacting serogroup among seropositive participants was Icterohaemorrhagiae, followed by Australis. However, the highest reacting MAT serogroup has limited correlation with the actual infecting serogroup and thus must be interpreted with caution [37,38]. Alternatively, correlation between highest reacting serovar and infecting serovar can increase as the time since infection increases, as cross-reactivity may decrease with time [39]. In order to definitively identify which *Leptospira* serovars are circulating in USVI, as well as which are causing human disease, bacterial isolates need to be obtained and characterized from acutely infected people and animals. It is likely that multiple *Leptospira* serovars are circulating in USVI, which is supported by isolates obtained in recently published animal surveillance in USVI [40,41], and that seropositive individuals detected were exposed to a variety of serovars through different exposure routes.

Our data combined with recent and previous publications suggest that most seropositive individuals detected were likely exposed in USVI. Based on the identification of three locally-acquired human cases of leptospirosis after the 2017 hurricanes [1], a fourth case in 2018 [42], and recent detection of *Leptospira* spp. DNA and isolates from wild and domestic animals [40,41,43], leptospirosis is endemic in USVI. Some seropositive participants could have been exposed to *Leptospira* spp. outside of USVI while traveling or residing elsewhere part-time. However, while the majority of seropositive participants traveled outside of USVI in the preceding year, few resided outside of USVI for part of the year, and neither travel nor living outside of USVI part of the year were associated with seropositivity.

The epidemiology and ecology of leptospirosis can vary widely by geographic location; therefore, it is essential to investigate and monitor the risk factors and drivers of infection for cases at a local level. Depending on the setting, *Leptospira* spp. infections can be driven by a variety and potential combination of factors such as poor sanitation, rodents and wildlife, livestock, rainfall and flooding, occupation, travel, and recreational activities. Aside from estimating the burden and distribution of leptospirosis in USVI, this investigation was the first attempt at identifying potential local risk factors for infection on the islands.

Historically, in most places leptospirosis cases tend to be most common among young and middle-aged adult males [18,24,44,45]. Our finding that increased age was independently associated with seropositivity is unsurprising in the context of a serosurvey; as age increases, there is increased opportunity over time for exposure or re-exposure. This association has also been seen in previous Caribbean serosurveys [30,31]. While acute cases tend to occur in young and middle-aged adults most often, anti-*Leptospira* antibodies can persist for several years, sometimes up to a decade or more [33,46]. Notably, two seropositive participants were children aged five and thirteen years. Although illness is more common in adults, children are susceptible to infection and disease [18,47–50], so leptospirosis should be considered when a child with potential exposure risks presents with an acute febrile illness. Our investigation did not include children under five years of age due to expected low seropositivity at this age and blood collection concerns, therefore the exposure prevalence among the youngest children in USVI is still unknown. While males were more commonly seropositive in this investigation, there was no significant difference from females.

Racial disparities in those affected by leptospirosis in USVI was identified as a potential problem worth investigating further. Self-identifying as full or partial white race was independently protective against seropositivity. This finding could be due to a variety of reasons, such as potential relationships between race and socioeconomic status or race and health disparities. People with lower socioeconomic status may be more likely to experience some common risk factors for leptospirosis including lower quality housing and neighborhoods with poorer infrastructure leading to more likely exposures to rodents, flood water, and sewage. A serosurvey in a crowded urban area in Brazil showed that both black race and socioeconomic status were independent predictors of *Leptospira* spp. exposure [51].

Two types of animal exposures were identified as independently associated with seropositivity by logistic regression, including contact with rodents or their body fluids and/or seeing them around the home or property, and contact with cows or their body fluids. Rodents, recognized as one of the main reservoirs for *Leptospira* spp. [6,17], can be a direct or indirect source of exposure in many environments, often playing an especially key role in human infections in urban areas [5,52], but also important in suburban and rural areas. Notably, all three post-hurricane leptospirosis cases in USVI had evidence of rodents documented in their home or work environments, among other potential sources of infection, and one had direct contact with rodent feces [1]. A recent investigation of rodent *Leptospira* spp. carriage in USVI identified leptospires in the kidneys of 45.7% of 140 rodents tested from the three main islands [41], which supports the findings in this investigation that rodent exposure is an important risk factor for human leptospirosis in USVI. *Leptospira* serovars most commonly associated with rodents include Icterohemorrhagiae (rats) and Ballum (mice) [5–7,53], which were also the serogroups isolated in the USVI rodent investigation [41]. Additionally, serogroup Icterohaemorrhagiae was the most common highest reacting serovar in this investigation, further supporting that rodents may be a significant source of *Leptospira* spp. exposure in USVI. Cows are another commonly recognized reservoir of *Leptospira* spp. and can contribute to human infections both through direct contact and environmental contamination with their urine. Recent leptospirosis testing of cows at an abattoir in St. Croix demonstrated 25% seropositivity among cows tested, and one cow that was shedding leptospires in the urine [43]. Further investigation is needed to draw stronger conclusions regarding the most important direct and indirect animal sources of human infection with *Leptospira* spp. in USVI, including identifying isolates from acute human leptospirosis cases and linking those to *Leptospira* species and serogroups identified from isolates found in animals on the islands, both in recent domestic animal and wildlife surveillance studies and future studies. Knowledge of the animal drivers of human infection can inform public health messaging and leptospirosis prevention methods.

Covering cisterns and other rainwater collection containers was significantly protective against seropositivity on bivariate analysis; this factor was not evaluated in the multivariate logistic regression model since many participants did not use cistern water or other collected rainwater as a drinking water source and would have been excluded from the model. A cover over these rainwater collection containers would help to prevent animals, animal excrement, and debris from entering and potentially contaminating the water, however, would not address animal urine running into the cistern with rainwater from the roof. *Leptospira* spp. were previously identified in cistern water associated with a post-hurricane case of leptospirosis in USVI [1], and a recent study of cistern water in USVI identified *Leptospira* spp. in 3/47 (6.4%) cisterns tested by PCR [54]. In Hawaii rainwater catchment systems were associated with leptospirosis cases and leptospires were identified in catchment rainwater [55]. Our findings provide further support for the hypothesis that untreated or inadequately treated cistern water could be an important potential source of human infection with *Leptospira* spp. in USVI and other endemic areas where cistern water and other collected rainwater is used as a source of drinking, bathing, cleaning, or farming water, such as in many other Caribbean islands and tropical and island areas [56]. Cistern water as a potential source of *Leptospira* spp. infection is a prime example of how the risk factors for leptospirosis can be dependent on the conditions and practices of specific localities and is especially important in USVI since almost every household has a cistern. Even though cisterns were often not used for drinking water by participants, many indicated contact with untreated cistern water, which could have been used for bathing, household chores, or farming.

Self-reported jaundice experienced after the 2017 hurricanes was significantly associated with seropositivity on bivariate analysis—it was not included in the multivariate logistic regression model because the intention was to analyze risk factors for exposure. Clinicians in USVI should include leptospirosis as a differential diagnosis for patients with acute febrile illness, especially if jaundice is present and/or after a hurricanes or flooding. While non-specific, jaundice is a common clinical sign of leptospirosis and is one of the two main components, along with renal disease, in the classically described presentation of severe leptospirosis called Weil’s disease [7,17].

Our finding of significantly increased seropositivity in urban areas should be interpreted cautiously as the classification of community type (rural, suburban, or urban) was determined subjectively by the survey team, and this association did not remain significant in multivariate logistic regression analysis. However, this finding makes sense in the context of rodent sightings and exposure being predictive of seropositivity, as contact with rodents and their urine may be more common in urban areas, especially those with poor infrastructure. If leptospirosis is more common in urban areas, this would provide a priority population for future public education campaigns.

Deer may be worth including in future animal studies to determine if they are commonly affected by leptospirosis in USVI and may act as a source of human infection, since seeing deer around the home or property was significantly associated with seropositivity, although this did not remain significant in our logistic regression model. Fishing as an occupation being significantly associated with seropositivity may be misleading in USVI, as there is very little fresh water on the islands and fishing is conducted in salt water, which is unlikely to be contaminated with viable leptospires, and this association did not persist in our logistic regression model. It is also possible that a confounding variable, such as rodent exposure related to fishing, may have led to the noted association. While seeing bats around the home or property had a protective effect against seropositivity, this association did not persist in our logistic regression model, and whether bats were seen was not uniformly asked, but was a common response to participants being asked if they had seen any types of wildlife around the home.

Our serosurvey investigation was designed to, and achieved the needed sample size, to provide a statistically representative estimate of leptospirosis seroprevalence across USVI. A multi-stage cluster analysis with random door-to-door visits in randomly selected communities ensured that a wide diversity of participants were enrolled. However, for safety reasons, participants were only enrolled during daylight hours, corresponding primarily to common work hours, and therefore there may have been a selection bias, highlighted by the fact that 67% of households visited were unavailable to field teams—weighted analysis was used to correct for such potential biases. For common free text “other” responses where noted, creating new response variables for analysis should be interpreted conservatively because they were not presented to all participants. Sampling a larger number of census blocks than originally planned likely increased the diversity of our selection pool, however sampling fewer than eight households in each census block may have biased our analyses towards the null hypotheses. Importantly, while this investigation established some associations between risk factors and seropositivity, further detailed investigation of acute leptospirosis cases, such as through active or enhanced passive surveillance, is needed to further solidify the most important risk factors for leptospirosis in USVI.

This public health surveillance investigation establishes that leptospirosis, a disease unreported in USVI before the 2017 hurricanes, is an important public health concern warranting further investigation and attention on the islands, and provides an initial description of potential local risk factors for exposure. Continued education of both the public and clinicians in USVI on leptospirosis will aid in timely illness identification and treatment in USVI residents and tourists to the islands, which may help decrease the severity and duration of disease, and will also contribute towards incorporating recommended infection prevention methods. Based on the post-hurricane leptospirosis cases identified, as well as the findings of this investigation, work is currently underway to build diagnostic capacity for leptospirosis on-island. While there is much still to learn about the specifics of the epidemiology and ecology of leptospirosis in USVI, this serosurvey has and will help to guide future human and animal studies as well as inform education campaigns in the interim. Ongoing leptospirosis disease surveillance and preparedness is essential in USVI and other Caribbean islands, as is enhanced surveillance after hurricanes, heavy rainfall, and floods. These findings also highlight the importance of conducting surveillance for leptospirosis in other locations throughout the world where the climate is conducive to *Leptospira* spp. but leptospirosis surveillance, diagnostics, and investigations have not previously been conducted.

## Supporting information

S1 Appendix USVI Leptospirosis Serosurvey Household Questionnaire. (DOCX)

S2 Appendix USVI Leptospirosis Serosurvey Individual Questionnaire. (DOCX)

S1 Table CDC’s *Leptospira* Microscopic Agglutination Test Antigen Panel for Serology Testing of Leptospirosis Serosurvey Participants in USVI, March 2019. (DOCX)

S2 Table *Leptospira* Microscopic Agglutination Test Highest Titer and Highest Reacting Serogroup(s) of Seropositive Participants in the Leptospirosis Serosurvey in USVI, March 2019. (DOCX)

## References

[1] Marinova-Petkova A, Guendel I, Strysko JP, Ekpo LL, Galloway R, Yoder J, et al. First Reported Human Cases of Leptospirosis in the United States Virgin Islands in the Aftermath of Hurricanes Irma and Maria, September-November 2017. Open forum infectious diseases. 2019;6(7):ofz261. doi: 10.1093/ofid/ofz261 31289729 PMC6602892

[2] Ahl AS, Miller DA, Bartlett PC. Leptospira serology in small ruminants on St. Croix, U.S. Virgin Islands. Annals of the New York Academy of Sciences. 1992;653:168–71. doi: 10.1111/j.1749-6632.1992.tb19640.x 1626866

[3] Picardeau M. Virulence of the zoonotic agent of leptospirosis: still terra incognita? Nature reviews Microbiology. 2017;15(5):297–307. doi: 10.1038/nrmicro.2017.5 28260786

[4] Costa F, Hagan JE, Calcagno J, Kane M, Torgerson P, Martinez-Silveira MS, et al. Global Morbidity and Mortality of Leptospirosis: A Systematic Review. PLoS neglected tropical diseases. 2015;9(9):e0003898. doi: 10.1371/journal.pntd.0003898 26379143 PMC4574773

[5] Goarant C. Leptospirosis: risk factors and management challenges in developing countries. Res Rep Trop Med. 2016;7:49–62. doi: 10.2147/RRTM.S102543 30050339 PMC6028063

[6] Ellis WA. Animal leptospirosis. Current topics in microbiology and immunology. 2015;387:99–137. doi: 10.1007/978-3-662-45059-8_6 25388134

[7] Levett PN. Leptospirosis. Clinical microbiology reviews. 2001;14(2):296–326. doi: 10.1128/CMR.14.2.296-326.2001 11292640 PMC88975

[8] Bierque E, Thibeaux R, Girault D, Soupé-Gilbert ME, Goarant C. A systematic review of Leptospira in water and soil environments. PloS one. 2020;15(1):e0227055. doi: 10.1371/journal.pone.0227055 31986154 PMC6984726

[9] Lau CL, Smythe LD, Craig SB, Weinstein P. Climate change, flooding, urbanisation and leptospirosis: fuelling the fire? Transactions of the Royal Society of Tropical Medicine and Hygiene. 2010;104(10):631–8. doi: 10.1016/j.trstmh.2010.07.002 20813388

[10] Sanders EJ, Rigau-Pérez JG, Smits HL, Deseda CC, Vorndam VA, Aye T, et al. Increase of leptospirosis in dengue-negative patients after a hurricane in Puerto Rico in 1996 [correction of 1966]. The American journal of tropical medicine and hygiene. 1999;61(3):399–404. doi: 10.4269/ajtmh.1999.61.399 10497979

[11] Naing C, Reid SA, Aye SN, Htet NH, Ambu S. Risk factors for human leptospirosis following flooding: A meta-analysis of observational studies. PloS one. 2019;14(5):e0217643. doi: 10.1371/journal.pone.0217643 31141558 PMC6541304

[12] Togami E, Kama M, Goarant C, Craig SB, Lau C, Ritter JM, et al. A Large Leptospirosis Outbreak following Successive Severe Floods in Fiji, 2012. The American journal of tropical medicine and hygiene. 2018;99(4):849–51. doi: 10.4269/ajtmh.18-0335 30141390 PMC6159581

[13] Dechet AM, Parsons M, Rambaran M, Mohamed-Rambaran P, Florendo-Cumbermack A, Persaud S, et al. Leptospirosis outbreak following severe flooding: a rapid assessment and mass prophylaxis campaign; Guyana, January-February 2005. PloS one. 2012;7(7):e39672. doi: 10.1371/journal.pone.0039672 22808049 PMC3392270

[14] Amilasan AS, Ujiie M, Suzuki M, Salva E, Belo MC, Koizumi N, et al. Outbreak of leptospirosis after flood, the Philippines, 2009. Emerging infectious diseases. 2012;18(1):91–4. doi: 10.3201/eid1801.101892 22257492 PMC3310081

[15] McClain JB, Ballou WR, Harrison SM, Steinweg DL. Doxycycline therapy for leptospirosis. Ann Intern Med. 1984;100(5):696–8. doi: 10.7326/0003-4819-100-5-696 6712032

[16] Brett-Major DM, Coldren R. Antibiotics for leptospirosis. Cochrane Database Syst Rev. 2012(2):Cd008264. doi: 10.1002/14651858.CD008264.pub2 22336839 PMC11299142

[17] Haake DA, Levett PN. Leptospirosis in humans. Current topics in microbiology and immunology. 2015;387:65–97. doi: 10.1007/978-3-662-45059-8_5 25388133 PMC4442676

[18] Bruce MG, Sanders EJ, Leake JA, Zaidel O, Bragg SL, Aye T, et al. Leptospirosis among patients presenting with dengue-like illness in Puerto Rico. Acta tropica. 2005;96(1):36–46. doi: 10.1016/j.actatropica.2005.07.001 16083836

[19] Neaterour P, Rivera A, Galloway RL, Negrón MG, Rivera-Garcia B, Sharp TM. Fatal Leptospira spp./Zika Virus Coinfection-Puerto Rico, 2016. The American journal of tropical medicine and hygiene. 2017;97(4):1085–7. doi: 10.4269/ajtmh.17-0250 28722594 PMC5637617

[20] Tomashek KM, Rivera A, Torres-Velasquez B, Hunsperger EA, Munoz-Jordan JL, Sharp TM, et al. Enhanced Surveillance for Fatal Dengue-Like Acute Febrile Illness in Puerto Rico, 2010–2012. PLoS neglected tropical diseases. 2016;10(10):e0005025. doi: 10.1371/journal.pntd.0005025 27727271 PMC5058557

[21] Brown MG, Vickers IE, Salas RA, Smikle MF. Leptospirosis in suspected cases of dengue in Jamaica, 2002–2007. Trop Doct. 2010;40(2):92–4. doi: 10.1258/td.2009.090340 20305103

[22] Lindo J, Brown PD, Vickers I, Brown M, Jackson ST, Lewis-Fuller E. Leptospirosis and malaria as causes of febrile illness during a dengue epidemic in Jamaica. Pathogens and global health. 2013;107(6):329–34. doi: 10.1179/2047773213Y.0000000112 24188242 PMC4001614

[23] McBride AJ, Athanazio DA, Reis MG, Ko AI. Leptospirosis. Curr Opin Infect Dis. 2005;18(5):376–86. doi: 10.1097/01.qco.0000178824.05715.2c 16148523

[24] Adesiyun AA, Baboolal S, Suepaul S, Dookeran S, Stewart-Johnson A. Human leptospirosis in the Caribbean, 1997–2005: characteristics and serotyping of clinical samples from 14 countries. Revista Panamericana de Salud Publica/Pan American Journal of Public Health. 2011;29(5):350–7. 21709940

[25] Peters A, Vokaty A, Portch R, Gebre Y. Leptospirosis in the Caribbean: a literature review. Revista panamericana de salud publica = Pan American journal of public health. 2017;41:e166. doi: 10.26633/RPSP.2017.166 31384278 PMC6650629

[26] Munoz-Zanzi C, Groene E, Morawski BM, Bonner K, Costa F, Bertherat E, et al. A systematic literature review of leptospirosis outbreaks worldwide, 1970–2012. Revista panamericana de salud publica = Pan American journal of public health. 2020;44:e78. doi: 10.26633/RPSP.2020.78 32684917 PMC7363284

[27] Pedersen K, Anderson TD, Maison RM, Wiscomb GW, Pipas MJ, Sinnett DR, et al. Leptospira Antibodies Detected in Wildlife in the USA and the US Virgin Islands. Journal of wildlife diseases. 2018. doi: 10.7589/2017-10-269 29715063

[28] Bureau USC. U.S. Census Detailed Crosstabulations 2010 [Available from: https://www2.census.gov/census_2010/10-Island_Areas_Detailed_Cross_Tabulations/Virgin_Islands/.

[29] Dikken H KE. Serological typing methods of leptospires. Methods in Microbiology. London: Academic Press; 1978. p. 259–307.

[30] Alexander AD, Benenson AS, Byrne RJ, Diaz Rivera RS, Evans LB, Gochenour WS, et al. LEPTOSPIROSIS IN PUERTO RICO. Zoonoses Res. 1963;2:152–227. 14170551

[31] Everard CO, Maude GH, Hayes RJ. Leptospiral infection: a household serosurvey in urban and rural communities in Barbados and Trinidad. Ann Trop Med Parasitol. 1990;84(3):255–66. doi: 10.1080/00034983.1990.11812465 2222028

[32] Wood H, Drebot MA, Dewailly E, Dillon L, Dimitrova K, Forde M, et al. Seroprevalence of seven zoonotic pathogens in pregnant women from the Caribbean. The American journal of tropical medicine and hygiene. 2014;91(3):642–4. doi: 10.4269/ajtmh.14-0107 24914001 PMC4155571

[33] Cumberland P, Everard CO, Wheeler JG, Levett PN. Persistence of anti-leptospiral IgM, IgG and agglutinating antibodies in patients presenting with acute febrile illness in Barbados 1979–1989. European journal of epidemiology. 2001;17(7):601–8. doi: 10.1023/a:1015509105668 12086073

[34] Blackmore D, Schollum L, Moriarty KJTNZmj. The magnitude and duration of titres of leptospiral agglutinins in human sera. 1984;97(749):83–6. 6583569

[35] Terpstra WJ, Ligthart GS, Schoone GJ. ELISA for the detection of specific IgM and IgG in human leptospirosis. Journal of General Microbiology. 1985;131(2):377–85. doi: 10.1099/00221287-131-2-377 3981131

[36] Edelweiss E, Mailloux MJIjoz. The curve of immunoglobulins in human leptospirosis. 1982;9(1):51. 7174234

[37] Levett PN. Usefulness of serologic analysis as a predictor of the infecting serovar in patients with severe leptospirosis. Clinical infectious diseases: an official publication of the Infectious Diseases Society of America. 2003;36(4):447–52. doi: 10.1086/346208 12567302

[38] Smythe LD, Wuthiekanun V, Chierakul W, Suputtamongkol Y, Tiengrim S, Dohnt MF, et al. The microscopic agglutination test (MAT) is an unreliable predictor of infecting Leptospira serovar in Thailand. The American journal of tropical medicine and hygiene. 2009;81(4):695–7. doi: 10.4269/ajtmh.2009.09-0252 19815889

[39] Katz AR, Effler PV, Ansdell VE. Comparison of serology and isolates for the identification of infecting leptospiral serogroups in Hawaii, 1979–1998. Trop Med Int Health. 2003;8(7):639–42. doi: 10.1046/j.1365-3156.2003.01071.x 12828547

[40] Cranford HM, Browne AS, LeCount K, Anderson T, Hamond C, Schlater L, et al. Mongooses (Urva auropunctata) as reservoir hosts of Leptospira species in the United States Virgin Islands, 2019–2020. PLoS neglected tropical diseases. 2021;15(11):e0009859. doi: 10.1371/journal.pntd.0009859 34780473 PMC8592401

[41] Hamond C, Browne AS, de Wilde LH, Hornsby RL, LeCount K, Anderson T, et al. Assessing rodents as carriers of pathogenic Leptospira species in the U.S. Virgin Islands and their risk to animal and public health. Sci Rep. 2022;12(1):1132. doi: 10.1038/s41598-022-04846-3 35064157 PMC8782869

[42] CDC. Nationally Notifiable Infectious Diseases and Conditions, United States: Annual Tables 2018 [Table 2h. Annual reported cases of notifiable diseases, by region and reporting area—Unites States and U.S. Territories, 2018]. Available from: https://wonder.cdc.gov/nndss/static/2018/annual/2018-table2h.html.

[43] Cranford HM, Taylor M, Browne AS, Alt DP, Anderson T, Hamond C, et al. Exposure and Carriage of Pathogenic Leptospira in Livestock in St. Croix, U.S. Virgin Islands. Trop Med Infect Dis. 2021;6(2). 10.3390/tropicalmed6020085 PMC8163180 34073665

[44] Katz AR, Buchholz AE, Hinson K, Park SY, Effler PV. Leptospirosis in Hawaii, USA, 1999–2008. Emerging infectious diseases. 2011;17(2):221–6. doi: 10.3201/eid1702.101109 21291592 PMC3204774

[45] Ko AI, Galvao Reis M, Ribeiro Dourado CM, Johnson WD Jr., Riley LW. Urban epidemic of severe leptospirosis in Brazil. Salvador Leptospirosis Study Group. Lancet. 1999;354(9181):820–5. 10485724 10.1016/s0140-6736(99)80012-9

[46] Rees EM, Lau CL, Kama M, Reid S, Lowe R, Kucharski AJ. Estimating the duration of antibody positivity and likely time of Leptospira infection using data from a cross-sectional serological study in Fiji. PLoS neglected tropical diseases. 2022;16(6):e0010506. doi: 10.1371/journal.pntd.0010506 35696427 PMC9232128

[47] Everard CO, Fraser-Chanpong GM. Serological evidence of leptospirosis in Caribbean schoolchildren. Transactions of the Royal Society of Tropical Medicine and Hygiene. 1979;73(5):591–3. doi: 10.1016/0035-9203(79)90062-2 531914

[48] Everard CO, Hayes RJ, Edwards CN. Leptospiral infection in school-children from Trinidad and Barbados. Epidemiology and infection. 1989;103(1):143–56. doi: 10.1017/s0950268800030442 2789146 PMC2249486

[49] Katz AR, Ansdell VE, Effler PV, Middleton CR, Sasaki DM. Leptospirosis in Hawaii, 1974–1998: epidemiologic analysis of 353 laboratory-confirmed cases. The American journal of tropical medicine and hygiene. 2002;66(1):61–70. doi: 10.4269/ajtmh.2002.66.61 12135270

[50] Biggs HM, Bui DM, Galloway RL, Stoddard RA, Shadomy SV, Morrissey AB, et al. Leptospirosis among hospitalized febrile patients in northern Tanzania. The American journal of tropical medicine and hygiene. 2011;85(2):275–81. doi: 10.4269/ajtmh.2011.11-0176 21813847 PMC3144825

[51] Reis RB, Ribeiro GS, Felzemburgh RD, Santana FS, Mohr S, Melendez AX, et al. Impact of environment and social gradient on Leptospira infection in urban slums. PLoS neglected tropical diseases. 2008;2(4):e228. doi: 10.1371/journal.pntd.0000228 18431445 PMC2292260

[52] Costa F, Zeppelini CG, Ribeiro GS, Santos N, Reis RB, Martins RD, et al. Household rat infestation in urban slum populations: Development and validation of a predictive score for leptospirosis. PLoS neglected tropical diseases. 2021;15(3):e0009154. doi: 10.1371/journal.pntd.0009154 33657101 PMC7959339

[53] Bharti AR, Nally JE, Ricaldi JN, Matthias MA, Diaz MM, Lovett MA, et al. Leptospirosis: a zoonotic disease of global importance. The Lancet Infectious diseases. 2003;3(12):757–71. doi: 10.1016/s1473-3099(03)00830-2 14652202

[54] Rao G, Kahler A, Voth-Gaeddert L, Cranford H, Libbey S, Galloway R, et al. Microbial Characterization, Factors Contributing to Contamination, and Household Use of Cistern Water, US Virgin Islands. ACS ES&T Water. 2022. doi: 10.1021/acsestwater.2c00389 PMC9745795 36530952

[55] Sasaki DM, Pang L, Minette HP, Wakida CK, Fujimoto WJ, Manea SJ, et al. Active surveillance and risk factors for leptospirosis in Hawaii. The American journal of tropical medicine and hygiene. 1993;48(1):35–43. doi: 10.4269/ajtmh.1993.48.35 8427386

[56] Khayan K, Heru Husodo A, Astuti I, Sudarmadji S, Sugandawaty Djohan T. Rainwater as a Source of Drinking Water: Health Impacts and Rainwater Treatment. J Environ Public Health. 2019;2019:1760950. doi: 10.1155/2019/1760950 31379953 PMC6657612

