# Supplementary material for: Seroprevalence, distribution, and risk factors for human leptospirosis in the United States Virgin Islands
Source: PLoS Negl Trop Dis. 2022 Nov 15;16(11):e0010880. doi: 10.1371/journal.pntd.0010880 (PMC9665390; doi:10.1371/journal.pntd.0010880)
Supplement: S1 Appendix — (DOCX) [file pntd.0010880.s001.docx]

**S1 Appendix. USVI Leptospirosis Serosurvey Household Questionnaire**

***Instructions for interviewer: Please read the questions as written. There are some introductory remarks to each section highlighted in grey. Please read these introductions to the participant.***

**FOR INTERVIEWER TO COMPLETE:**

**Interviewer Initials: __________ Date: ____ / ____ / ____ (mm /dd /yy)**  **Household ID: _____________**

1. **Island:**  STX  STT  STJ
2. **Census Block #: _________**
3. **GPS (handheld) Coordinates:** Lat: ________________________ Lon: ________________________
4. **Community classification:**  Rural  Urban  Suburban
5. **Type of residence:**  Single-family house  Apartment  Other *(specify*): _______________
6. **How many people live in the household? ___________**  Participant prefers not to say
7. **How many are ≥5 years and live in V.I. ≥ 6 months per year? _____**  Participant prefers not to say
8. **Based on the consent / assent, how many eligible people refused participation?** ____________

| **Script 1: Thank you for agreeing to participate. We are going to ask you a few questions about your household. A household is those people currently living under the same roof.** |
| --- |

1. **What is the address where you receive your mail?**  Prefer not to say
2. **Street Number / Name: _________________ OR PO BOX: __________________**
3. **Zip Code – City: _____________________**
4. **What is the address of this home?**

 Same as mailing address  Prefer not to say

***If different from mailing address:***

1. **Street Number / Name: __________________________________**
2. **Zip Code – City: _____________________**

| **Script 2: Now we are going to ask you about your household water supply.** |
| --- |

1. **Which of the following water sources does your household use for drinking? Which is your primary source of drinking water?**
   1. **Locally bottled water (ex. Blue Mountain, Crystal Springs)**

 Yes, primary  Yes  No  Don’t know  Prefer not to say

- 1. **Stateside bottled water (ex. Dasani, Aquafina)**

 Yes, primary  Yes  No  Don’t know  Prefer not to say

- 1. **Piped city water (WAPA)**

 Yes, primary  Yes  No  Don’t know  Prefer not to say

- 1. **Personal well**

 Yes, primary  Yes  No  Don’t know  Prefer not to say

- 1. **Cistern**

 Yes, primary  Yes  No  Don’t know  Prefer not to say

- 1. **(Non-cistern) Drum, or any container collecting rainwater for household use**

 Yes, primary  Yes  No  Don’t know  Prefer not to say

- 1. **Other**

 Yes, primary  Yes  No  Don’t know  Prefer not to say

***If Yes, primary or Yes:***

Please specify the other water source your household uses for drinking: ____________________

1. **If cistern, non-cistern drum, or other is a drinking water source (primary source or not) in question 11: Does your cistern, non-cistern drum, or other drinking water container have the following?**
   1. **A cover to protect from rodents/debris**

 Yes  No  Don’t know  Prefer not to say

- 1. **A filtration system**

 Yes  No  Don’t know  Prefer not to say

1. **If cistern, non-cistern drum, or other is a drinking water source (primary source or not) in Question 11: Do you use any of the following to treat your cistern, non-cistern drum or other drinking water source?**
   1. **Boiling**

 Yes  No  Don’t know  Prefer not to say

- 1. **Chlorination**

 Yes  No  Don’t know  Prefer not to say

- 1. **Ultraviolet (UV light)**

 Yes  No  Don’t know  Prefer not to say

- 1. **Other**

 Yes, specify: ____________________  No  Don’t know  Prefer not to say

1. **If there is heavy rain, does your house or the land around your house ever flood?**

 Yes  No  Don’t know  Prefer not to say

- 1. **If yes, has this happened in the past year?**

 Yes  No  Don’t know  Prefer not to say

| **Script 3: Because leptospirosis is spread through animal urine, we are going to ask you some questions about animals your household owns.** |
| --- |

1. **In the past year, have any of the following animals lived inside your home or on the property around your home?**
   1. **Dogs**

 Yes  No  Don’t know  Prefer not to say

- 1. **Cows**

 Yes  No  Don’t know  Prefer not to say

- 1. **Pigs**

 Yes  No  Don’t know  Prefer not to say

- 1. **Horses/donkeys**

 Yes  No  Don’t know  Prefer not to say

- 1. **Goats/sheep**

 Yes  No  Don’t know  Prefer not to say

- 1. **Wildlife**

 Yes, specify: ____________________  No  Don’t know  Prefer not to say

| **Script 4. Now we are going to ask you questions about ways in which your household was affected by hurricanes Irma and Maria in September 2017.** |
| --- |

1. **Did your home or the land around your home experience flooding from the hurricanes?**

 Yes  No  Don’t know  Prefer not to say

1. **Was your home damaged from the hurricanes? For example, broken walls, roof, or windows.**

 Yes  No  Don’t know  Prefer not to say

1. **Did the hurricanes change your household’s drinking water sources?**

 Yes  No  Don’t know  Prefer not to say

1. **If yes, what were your sources of water until it was repaired? (*select all that apply*)**

 Locally bottled water

 Stateside bottled water

 Piped city water (WAPA)

 Personal well

 Cistern

 Non-cistern drums, or any container collecting rainwater

 Other, specify________________

 Don’t know

 Prefer not to say

| **Script 5. This is the end of the household survey. Thank you for answering all those questions. Next, we will ask each person who is participating in this survey some questions about themselves.** |
| --- |

**Notes:**

|  |
| --- |
