# Supplementary material for: Seroprevalence, distribution, and risk factors for human leptospirosis in the United States Virgin Islands
Source: PLoS Negl Trop Dis. 2022 Nov 15;16(11):e0010880. doi: 10.1371/journal.pntd.0010880 (PMC9665390; doi:10.1371/journal.pntd.0010880)
Supplement: S2 Appendix — (DOCX) [file pntd.0010880.s002.docx]

**S2 Appendix. USVI Leptospirosis Serosurvey Individual Questionnaire**

***Instructions for interviewer: Please read the questions as written. There are some introductory remarks to each section highlighted in grey. Please read these introductions to the participant.***

**FOR INTERVIEWER TO COMPLETE:**

**Interviewer Initials: __________ Date: _____ / _____ / _____ (mm/dd/yy)** **Participant ID: ______________**

**Participant verbal consent or assent obtained?**  **Yes**

**Parent/guardian verbal permission received?**  **Yes**   **Not applicable**

| **Script 1. Thank you for agreeing to participate. First, I will ask you a few questions about yourself, your health and travel history.** |
| --- |

1. **What is your first and last name?**
   1. **First Name: _____________________ b. Last name: ____________________**  Prefer not to say
2. **What is your gender?**  Male  Female  Other, specify: ___________  Prefer not to say
3. **What is your birthdate?** Month: _________ Day: ______ Year: _________  Prefer not to say
   1. ***If the participant prefers not to say, the interviewer should select one*:**  Adult  Child
4. **What is your race? Select one or more racial or ethnic group you use to describe yourself.**

 American Indian or Alaska Native

 Black or African American
 White/Caucasian

 Asian

 Native Hawaiian/Pacific Islander

 Hispanic or Latino

 Other race or ethnicity, specify: _______________________________

 Don’t know

 Prefer not to say

1. **What is your job? (*select all that apply*)**

 Office worker (Spend most of the workday in an office / at a computer)

 Healthcare provider (ex. a doctor, nurse, caregiver)

 Veterinarian, vet technician / assistant, or kennel staff

 Law enforcement officer or firefighter

 Slaughterhouse worker or meat inspector

 Maintenance, repair, construction, or building/grounds cleaning

 Farming crops

 Farming livestock (animals)

 Fishing

 Tourism/hospitality

 Forestry/ park worker/ ranger

 Plumber/sewer worker

 Garbage collector

 Student

 Unemployed or retired

 Other: _____________________________________________

 Prefer not to say

1. **How long have you lived in V.I.? _________ year(s)**  Prefer not to say
2. **Do you live outside of V.I. for part of the year?**  Yes  No  Prefer not to say
   1. **If yes, how many months per year on average do you spend living *in* V.I.?** _____  Prefer not to say
3. **In the past year, have you traveled outside of V.I.?**  Yes  No  Prefer not to say
   1. **If yes, where did you travel? ______________________________**  Prefer not to say
4. **Since the hurricanes in September 2017:**
   1. **Did you have a flu-like illness (ex. fever, chills, muscle/joint aches, headache, or nausea)?**

 Yes  No  Don’t know  Prefer not to say

- 1. **Did you have jaundice (yellowing of eyes, gums, or skin)?**

 Yes  No  Don’t know  Prefer not to say

- 1. **Were you diagnosed with kidney failure?**

 Yes  No  Don’t know  Prefer not to say

- 1. **Were you hospitalized for being sick?**

 Yes  No  Don’t know  Prefer not to say

| **Script 2. Because leptospirosis can be spread through urine-contaminated water and mud, I am going to ask you some questions about your contact with different types of water and mud in your day-to-day life.** |
| --- |

1. **In the past year, have you done any of the following activities that may have put you in contact with fresh water, wastewater, or mud?**
   1. **Bathing, wading, swimming, walking barefoot**

 Yes  No  Don’t know  Prefer not to say

- 1. **Boating, canoeing, kayaking**

 Yes  No  Don’t know  Prefer not to say

- 1. **Hiking**

 Yes  No  Don’t know  Prefer not to say

- 1. **Gardening, farming**

 Yes  No  Don’t know  Prefer not to say

- 1. **Other**

 Yes: _______________________  No  Don’t know  Prefer not to say

1. **If “yes” in question 10, have you had contact with any of the following types of water in the past year?**
   1. **River or stream (running water)**

 Yes  No  Don’t know  Prefer not to say

- 1. **Lake or pond (still water)**

 Yes  No  Don’t know  Prefer not to say

- 1. **Untreated cistern water**

 Yes  No  Don’t know  Prefer not to say

- 1. **Marsh or swamp**

 Yes  No  Don’t know  Prefer not to say

- 1. **Mud/wet soil**

 Yes  No  Don’t know  Prefer not to say

- 1. **Sewage water or run-off**

 Yes  No  Don’t know  Prefer not to say

- 1. **Marsh or swamp**

 Yes  No  Don’t know  Prefer not to say

- 1. **Other**

 Yes: _______________________  No  Don’t know  Prefer not to say

| **Script 3. Because leptospirosis can be spread by infected animals, I am going to ask you some questions about your contact with animals in your day-to-day life.** |
| --- |

1. **In the past year, have you ever had contact with any of the following animals? Animal contact includes feeding, grooming, milking, cleaning up areas where animals are housed, and contact with bodily fluids such as blood, urine, and reproductive fluids.**
   1. **Dogs**

 Yes  No  Don’t know  Prefer not to say

- 1. **Cows**

 Yes  No  Don’t know  Prefer not to say

- 1. **Pigs**

 Yes  No  Don’t know  Prefer not to say

- 1. **Horses/donkeys**

 Yes  No  Don’t know  Prefer not to say

- 1. **Goats/sheep**

 Yes  No  Don’t know  Prefer not to say

- 1. **Wildlife**

 Yes _____________________  No  Don’t know  Prefer not to say

1. **In the past year, have you seen rodents, or evidence of rodents (such as droppings, burrows, or holes) inside your house or around your house or land?**

 Yes  No  Don’t know  Prefer not to say

| **Script 4. Now, I am going to ask you some questions about your activities in the three months after hurricanes Maria and Irma struck V.I. (September to December 2017).** |
| --- |

1. **Did you continue to live in your home after the hurricanes?**

 Yes  No  Prefer not to say

- 1. **If no, did you visit or work on your home while it was flooded or damaged?**

 Yes  No  Prefer not to say  Not applicable

1. **Did you wade or swim in floodwater, or otherwise touch floodwater with your bare hands or feet?**

 Yes  No  Don’t know  Prefer not to say

1. **Were you involved in cleaning up debris following the hurricanes?**

| ***Script 5. Now, we have just a couple of more questions before the end of the survey.*** |
| --- |

 Yes  No  Don’t know  Prefer not to say

1. **We will send your test results to your mailing address, email, or phone.**
   1. **Is the household mailing address where you prefer to get your mail?**

 Same as household mailing address  Different from household address  Prefer not to say

***If different from household address:***

**Where do you prefer we mail your test results?**

**Street Number / Name: _________________ OR PO BOX: ___________________
Zip Code – City: _____________________**

- 1. **What is your email address? ____________________________________**  Prefer not to say
  2. **What is your phone number? ___________________________________**  Prefer not to say

1. **Can we contact you in the future for follow up related to this project, such as cistern water testing or if we have additional questions?**

 Yes  No

1. **Do we have your permission to test your blood sample in the future for other infectious diseases? This could help us understand other diseases that may be in USVI such as dengue. We will not test for STDs. Your blood sample and test results will be kept private. If additional infectious disease testing is done, test results will not be provided.**

| **Script 6. This is the end of the survey. Thank you for answering our questions and giving us your time.** |
| --- |

 Yes  No

**Notes:**

|  |
| --- |
