# Supplementary material for: Seroprevalence, distribution, and risk factors for human leptospirosis in the United States Virgin Islands
Source: PLoS Negl Trop Dis. 2022 Nov 15;16(11):e0010880. doi: 10.1371/journal.pntd.0010880 (PMC9665390; doi:10.1371/journal.pntd.0010880)
Supplement: S1 Table — (DOCX) [file pntd.0010880.s003.docx]

**S1 Table. CDC’s *Leptospira* Microscopic Agglutination Test Antigen Panel for Serology Testing of Leptospirosis Serosurvey Participants in USVI, March 2019**

| **Serogroup** | **Serovar** | **Strain** |
| --- | --- | --- |
| Australis | Australis | Ballico |
| Australis | Bratislava | Jez Bratislava |
| Autumnalis | Autumnalis | Akiyami A |
| Ballum | Ballum | Mus 127 |
| Bataviae | Bataviae | Van Tienen |
| Canicola | Canicola | Ruebush |
| Celledoni | Celledoni | Celledoni |
| Cynopteri | Cynopteri | 3522 C |
| Djasiman | Djasiman | Djasiman |
| Grippotyphosa | Grippotyphosa | No strain name |
| Hebdomadis | Borincana | HS 622 |
| Icterohaemorrhagiae | Icterohaemorrhagiae | RGA |
| Icterohaemorrhagiae | Mankarso | Mankarso |
| Javanica | Javanica | Veldrat Batavia 46 |
| Mini | Georgia | LT 117 |
| Pomona | Pomona | Pomona |
| Pyrogenes | Alexi | HS 616 |
| Pyrogenes | Pyrogenes | Salinem |
| Sejroe | Wolffi | 3705 |
| Tarassovi | Tarassovi | Perepelitsin |
