# Supplementary material for: Seroprevalence, distribution, and risk factors for human leptospirosis in the United States Virgin Islands
Source: PLoS Negl Trop Dis. 2022 Nov 15;16(11):e0010880. doi: 10.1371/journal.pntd.0010880 (PMC9665390; doi:10.1371/journal.pntd.0010880)
Supplement: S2 Table — (DOCX) [file pntd.0010880.s004.docx]

**S2 Table. *Leptospira* Microscopic Agglutination Test Highest Titer and Highest Reacting Serogroup(s) of Seropositive Participants in the Leptospirosis Serosurvey in USVI, March 2019**

| Sample No. | Highest Titer | Highest Titer Reacting Serogroup(s) |
| --- | --- | --- |
| 1 | 100 | Djasiman |
| 2 | 200 | Autumnalis |
| 3 | 200 | Pyrogenes |
| 4 | 800 | Djasiman |
| 5 | 3200 | Pyrogenes |
| 6 | 200 | Australis |
| 7 | 100 | Tarrasovi |
| 8 | 100 | Australis, Icterohaemorrhagiae |
| 9 | 200 | Icterohaemorrhagiae |
| 10 | 200 | Icterohaemorrhagiae |
| 11 | 1600 | Australis |
| 12 | 100 | Canicola, Icterohaemorrhagiae |
| 13 | 200 | Canicola |
| 14 | 200 | Sejroe |
| 15 | 200 | Canicola |
| 16 | 100 | Bataviae |
| 17 | 400 | Pyrogenes |
| 18 | 200 | Icterohaemorrhagiae |
| 19 | 100 | Canicola |
| 20 | 100 | Tarrasovi |
| 21 | 100 | Autumnalis, Australis |
| 22 | 100 | Australis |
| 23 | 100 | Icterohaemorrhagiae |
| 24 | 100 | Icterohaemorrhagiae |
| 25 | 100 | Bataviae, Australis, Canicola, Icterohaemorrhagiae |
| 26 | 200 | Pyrogenes |
| 27 | 200 | Tarrasovi |
| 28 | 200 | Icterohaemorrhagiae |
| 29 | 100 | Canicola |
| 30 | 1600 | Ballum |
| 31 | 100 | Australis |
| 32 | 100 | Australis |
| 33 | 100 | Canicola |
| 34 | 100 | Icterohaemorrhagiae |
| 35 | 100 | Icterohaemorrhagiae |
| 36 | 100 | Icterohaemorrhagiae |
| 37 | 100 | Icterohaemorrhagiae |
| 38 | 200 | Australis, Icterohaemorrhagiae |
| 39 | 400 | Icterohaemorrhagiae |
| 40 | 200 | Australis |
| 41 | 200 | Australis, Icterohaemorrhagiae |
| 42 | 800 | Icterohaemorrhagiae |
| 43 | 800 | Icterohaemorrhagiae |
| 44 | 200 | Icterohaemorrhagiae |
| 45 | 100 | Icterohaemorrhagiae, Pyrogenes |
| 46 | 100 | Icterohaemorrhagiae, Tarrasovi |
| 47 | 200 | Australis |
